# Correlation between the γ passing rates of IMRT plans and the volumes of air cavities and bony structures in head and neck cancer

**DOI:** 10.1186/s13014-021-01861-y

**Published:** 2021-07-21

**Authors:** Zhengwen Shen, Xia Tan, Shi Li, Xiumei Tian, Huanli Luo, Ying Wang, Fu Jin

**Affiliations:** Department of Radiation Oncology, Chongqing University Cancer Hospital, Chongqing, 400030 China

**Keywords:** IMRT QA, γ passing rates, Air cavities, Bony structures, Monte Carlo

## Abstract

**Background:**

Both patient-specific dose recalculation and γ passing rate analysis are important for the quality assurance (QA) of intensity modulated radiotherapy (IMRT) plans. The aim of this study was to analyse the correlation between the γ passing rates and the volumes of air cavities (*V*_air_) and bony structures (*V*_bone_) in target volume of head and neck cancer.

**Methods:**

Twenty nasopharyngeal carcinoma and twenty nasal natural killer T-cell lymphoma patients were enrolled in this study. Nine-field sliding window IMRT plans were produced and the dose distributions were calculated by anisotropic analytical algorithm (AAA), Acuros XB algorithm (AXB) and SciMoCa based on the Monte Carlo (MC) technique. The dose distributions and γ passing rates of the targets, organs at risk, air cavities and bony structures were compared among the different algorithms.

**Results:**

The γ values obtained with AAA and AXB were 95.6 ± 1.9% and 96.2 ± 1.7%, respectively, with 3%/2 mm criteria (*p* > 0.05). There were significant differences (*p* < 0.05) in the γ values between AAA and AXB in the air cavities (86.6 ± 9.4% vs. 98.0 ± 1.7%) and bony structures (82.7 ± 13.5% vs. 99.0 ± 1.7%). Using AAA, the γ values were proportional to the natural logarithm of *V*_air_ (R^2^ = 0.674) and inversely proportional to the natural logarithm of *V*_bone_ (R^2^ = 0.816). When the *V*_air_ in the targets was smaller than approximately 80 cc or the *V*_bone_ in the targets was larger than approximately 6 cc, the γ values of AAA were below 95%. Using AXB, no significant relationship was found between the γ values and *V*_air_ or *V*_bone_.

**Conclusion:**

In clinical head and neck IMRT QA, greater attention should be paid to the effect of *V*_air_ and *V*_bone_ in the targets on the γ passing rates when using different dose calculation algorithms.

## Introduction

Radiation dose escalation has been shown to be beneficial for local control and improving overall survival in the treatment of cancer [1, 2]. However, these benefits may be accompanied by higher incidences of acute and late toxicity [3, 4]. Intensity modulated radiation therapy (IMRT) results in desirable target coverage and toxicity reduction to organs at risk (OARs), but it is associated with many uncertainties leading to dose deviations that affect predictions about tumour control probability (TCP) and normal tissue complication probability (NTCP). The American Association of Physicists in Medicine (AAPM) Task Group 40 report (TG-40) recommends that the dose delivered to patients should be within 5% of the prescribed dose [5], but such an accurate and consistent dose delivery is complicated, since many steps are involved during the treatment process; therefore, the dose deviations produced at each step should be as small as possible. Continually updated guidelines have been provided to assure the accuracy of radiation treatment [6–12]; most focus on device evaluations and dose measurements, but few address the accuracy of the dose calculation algorithms. The human body is composed of different components, which increases the challenge of accurate calculation. Hence, in the quality assurance (QA) of the treatment planning system (TPS), the evaluation of the accuracy of the dose distribution produced by the TPS is indispensable.

Nasopharyngeal carcinoma (NPC) and nasal natural killer T-cell lymphoma (NNKTCL) are both characterized by regional and ethnic differences; the two cancers are more common in Eastern Asia than in Western countries, demonstrating a particularly high incidence in southern China [13, 14]. NPC and NNKTCL represent the major head and neck cancers commonly treated by IMRT. Respiratory and organ movement have little impact on setup errors for IMRT in head and neck cancer. Given the good performance of the equipment and correct operation by the therapists, dose deviations are thus mainly caused by the dose calculation algorithms. The target region for NPC and NNKTCL includes a considerable number of air cavities and bony structures, resulting in three heterogeneous interfaces: air–tissue, bone–tissue and air-bone. Radiation beams passing through these heterogeneous interfaces always lead to the electronic disequilibrium effect and dose perturbations.

To our knowledge, patient-specific dose recalculation using the Monte Carlo (MC) algorithm, as a step of IMRT QA, for head and neck cancer has not been investigated [15–17]. Therefore, one of our aims was to implement this QA step in real patients with NPC and NNKTCL using the MC method. Numerous studies have investigated the assessment of dose perturbations at heterogeneous interfaces [18–20]; however, studies that compared the results produced by TPS against measured data in heterogeneous media have typically focused on the agreement in the area around the heterogeneity, and only a few have investigated the agreement directly within the heterogeneity. Therefore, another aim of our study was to compare the dosimetric difference directly inside air cavities and bony structures based on the QA results and analyse the correlation between the γ passing rates and the volume of the air cavities (*V*_air_) and bony structures (*V*_bone_).

## Materials and methods

### Patients and prescription

Twenty NPC patients and twenty NNKTCL patients were selected from the clinical database. All patients underwent CT scans with a 3 mm slice thickness. Three planning target volumes were defined for each NPC patient with 70.4 Gy prescribed to PGTVnx and PGTVnd, 60.8 Gy to PTV1, and 54.4 Gy to PTV2 in 32 daily fractions. For each NNKTCL patient, the prescribed PTV dose was 56 Gy in 28 daily fractions.

The volumes of the contoured PGTVnx for NPC and the PTV for NNKTCL included a considerable number of air cavities and bony structures. To assess the dosimetric impact on these volumes, the air cavities and bony structures included in PGTVnx and PTV were contoured separately. Since PGTVnx contained the largest proportion of air cavities and bony structures relative to the other targets with lower dose prescriptions among the NPC patients, the analysis was confined to PGTVnx in this study.

### Treatment planning

All plans were generated using 6 MV photon beams and modulated with a Millennium 120 multi-leaf collimator (MLC) from a Varian Clinac IX (Varian Medical Systems, Palo Alto, California, USA) in Eclipse TPS version 15.6. The plans were created using nine fields that were evenly distributed in coplanar directions with the sliding window technique. Because the lenses and optic nerves were close to the PTV for NNKTCL patients, the angle of the collimator and the position of the jaws in some fields were adjusted, and fixed jaws were used during optimization.

The optimization goal was to ensure that at least 95% of the volume of the targets received the prescribed dose and that the maximal dose of the targets would not exceed 110% of the prescribed dose, while minimizing the doses to the OARs, whose dose constraints are given in Table 1. After optimization, dose calculations were performed using anisotropic analytical algorithm (AAA) version 15.6.06 with a 2.5 mm grid size. The quality of each plan was assessed with regard to its clinical acceptability by oncologists. Each plan was subsequently recalculated using Acuros XB algorithm (AXB) version 15.6.06 (dose to medium), using the same calculation settings as AAA.

**Table 1:** Dose constraints for the organs at risk

OARs	Constraints	OARs	Constraints
Brainstem	*D*_max_<54Gy	Spinal cord	*D*_max_<40Gy
Optic chiasm	*D*_max_<54Gy	Optic nerves	*D*_max_<54Gy
Pituitary	*D*_max_<54Gy	Eyes	*D*_mean_<25Gy
Temporal lobes	*V*_60_<2cc	Parotid glands	*D*_mean_<30Gy
Lens	*D*_max_<12Gy	Inner ears	*D*_mean_<35Gy
Oral cavity	*D*_mean_<38Gy	Mandible	*V*_55_<10%

### SciMoCa model and dose recalculation

The SciMoCa algorithm for linear accelerators is described in detail in Ref. [21, 22]. It combines the concepts of the voxel-based Monte Carlo algorithm with some element of EGSnrc [23]. The treatment head simulation, employing five virtual sources determined from BEAMnrc, is an evolution from previous models [24, 25]. The 6 MV beam modality of the Varian Clinac IX with the Millennium 120 MLC was commissioned using the same measurement data used to commission the Eclipse TPS. The accelerator head was commissioned on the basis of depth dose curves, profile curves measured at five depths (1.5, 5, 10, 20, 30 cm) and output factors for square fields (3 × 3, 5 × 5, 10 × 10, 15 × 15, 20 × 20, 30 × 30, 40 × 40 mm^2^). The dosimetry leaf gap and leaf transmission of the MLC were configured to match the measured data [26].

The selected plans were exported to SciMoCa and recalculated using the DICOM images, structure sets and plan information. The dose was reported as dose to medium. The grid size of 2.5 mm used for the calculation was the same as that of TPS. SciMoCa can employ uncertainty levels of 2%, 1%, and 0.5%. Smaller the statistical uncertainty is, the more accurate the MC calculation. To obtain the most accurate QA results, the 0.5% statistical uncertainty level was used in our study.

### Dosimetric evaluation and data analysis

For the forty real patient plans (twenty NPC and twenty NNKTCL plans), the dosimetric parameters mentioned in Table 1 were compared. The results of the calculation from MC were used as the reference data sets, and the results of the calculation from AAA and AXB were used as the evaluated data sets. The dose distributions from AAA and AXB were compared with those of MC using a global γ evaluation with suppression of doses below 10% of the maximum dose. The percentage of points fulfilling the γ evaluation was scored as the γ passing rate. It has been recommended that the γ passing rates should be ≥ 95% with a dose difference of 3% and a distance to agreement of 2 mm (3%/2 mm) [12]. The γ passing rates were scored for the entire plan and for the targets and OARs. The mean dose to the air cavities and bony structures in the targets estimated by AAA and AXB were calculated and compared with that estimated by MC for each patient. The γ passing rates were also scored separately for the air cavities and bony structures. Scatter plots were used to explore the correlation between the γ passing rates and *V*_air_/*V*_bone_.

The paired *t*-test was used to determine if there was a significant difference for each of the parameters. *p* < 0.05 was considered statistically significant. SPSS statistical software (SPSS, Chicago, IL, USA) was used for all analyses.

## Results

### γ evaluation results

Table 2 summarizes the γ passing rates for forty clinical head and neck cancer patients using AAA and AXB for the entire plan and for the targets sand OARs. The γ passing rates from AXB were higher than those from AAA. The γ values from AAA for the entire plan and for the targets and OARs were over 95%, except for PGTVnx and PTV. Table 2 also shows that the γ evaluation results from AAA and AXB for PGTVnx, PTV1, PTV, mandible and oral cavity were statistically significant.

**Table 2:** γ passing rates for entire plans, targets and OARs. (%)

	AAA	AXB	*p *value
Entire plans	95.6±1.9	96.2±1.7	>0.05
PGTVnx	93.8±2.3	99.6±0.2	<0.05
PGTVnd	98.9±1.1	99.3±0.7	>0.05
PTV1	97.3±1.3	99.7±0.2	<0.05
PTV2	98.2±0.8	98.9±0.9	>0.05
PTV	90.3±6.2	97.2±5.3	<0.05
Brain stem	99.9±0.3	99.9±0.2	>0.05
Spinal cord	96.9±2.9	97.0±3.2	>0.05
Optic chiasm	99.9±0.2	99.9±0.2	>0.05
Left optic nerve	100±0	100±0	>0.05
Right optic nerve	99.9±0.4	100±0	>0.05
Pituitary	99.7±0.3	100±0	>0.05
Left len	99.5±0.3	99.7±0.2	>0.05
Right len	99.6±0.2	99.9±0.2	>0.05
Left eye	99.3±2.6	99.3±2.3	>0.05
Right eye	99.3±2.1	99.3±2.1	>0.05
Left temporal lobe	99.8±0.1	99.9±0.1	>0.05
Right temporal lobe	99.8±0.4	99.9±0.2	>0.05
Mandible	98.3±0.7	99.8±0.4	<0.05
Left parotid gland	99.0±1.5	99.6±0.6	>0.05
Right parotid gland	99.4±0.9	99.7±0.4	>0.05
Left inner ear	99.7±0.6	100±0	>0.05
Right inner ear	99.7±0.6	100±0	>0.05
Oral cavity	96.7±0.8	99.2±0.5	<0.05

### Dosimetric comparison

Table 3 summarizes the dose parameters of the targets and OARs and the number of plans satisfying the clinical requirements. The dose estimated by AXB provided better agreement with MC than AAA. AXB estimated a 0.1% ~ 1.5% lower target coverage and a 1.6 ~ 3.5 Gy higher target *D*_max_ than AAA. The *D*_max_ estimated by AXB to the serial organs, including the brain stem, spinal cord, optic chiasm, optic nerves, lens and pituitary was 0.1 ~ 0.7 Gy higher than that estimated by AAA. Using AXB, the estimated mean doses to the eyes, parotid glands, inner ears and oral cavity were 0.1 ~ 0.4 Gy lower than those estimated by AAA. *V*_60_ for the temporal lobes and *V*_55_ for the mandible provided by the three different methods were the same. The number of plans satisfying the clinical requirements calculated by AXB was no more than that calculated by AAA.

**Table 3:** Summary of the doses to the targets and OARs estimated by AAA, AXB and MC

Structure	Parameter	AAA	AXB	MC
PGTVnx	*V*_70.4_, %	97.9±2.3 (19)	96.7±2.9 (17)	96.7±3.0 (16)
	*D*_max_, Gy	75.8±1.4 (20)	77.4±1.4 (15)	77.4±1.5 (13)
PGTVnd	*V*_70.4_, %	97.3±1.1 (20)	96.7±2.4 (17)	96.6±2.8 (17)
	*D*_max_, Gy	75.6±1.5 (20)	77.2±1.3 (20)	77.6±1.2 (15)
PTV1	*V*_60.8_, %	95.9±1.5 (18)	95.8±1.3 (17)	95.6±1.5 (17)
PTV2	*V*_54.4_, %	95.5±2.3 (15)	94.7±2.2 (13)	94.1±2.4 (11)
PTV	*V*_56_, %	94.6±2.6 (17)	92.1±4.5 (16)	90.9±6.9 (15)
	*D*_max_, Gy	61.3±2.5 (15)	64.8±4.1 (13)	65.5±5.5 (12)
Brain stem	*D*_max_, Gy	46.9±13.8 (36)	47.2±13.8 (35)	47.5±14.1 (35)
Spinal cord	*D*_max_, Gy	32.7±11.4 (39)	32.8±11.4 (39)	33.2±11.3 (38)
Optic chiasm	*D*_max_, Gy	48.4±13.3 (40)	48.5±13.1 (40)	49.0±13.3 (39)
Left optic nerve	*D*_max_, Gy	43.2±16.0 (38)	43.4±16.1 (36)	43.4±16.2 (36)
Right optic nerve	*D*_max_, Gy	41.0±16.0 (38)	41.2±16.2 (38)	41.5±16.2 (38)
Pituitary	*D*_max_, Gy	54.8±9.8 (27)	55.4±9.6 (26)	56±9.9 (26)
Left len	*D*_max_, Gy	9.0±2.7 (37)	9.7±3.0 (36)	9.8±2.5 (35)
Right len	*D*_max_, Gy	11.2±10.5 (35)	11.5±10.5 (33)	11.8±11.1 (33)
Left eye	*D*_mean_, Gy	12.8±4.2 (40)	12.6±4.2 (40)	12.4±4.3 (40)
Right eye	*D*_mean_, Gy	13.6±5.6 (40)	13.4±5.6 (40)	13.2±5.7 (40)
Left temporal lobe	*V*_60_, cc	0.3±0.7 (37)	0.3±0.8 (37)	0.3±0.8 (37)
Right temporal lobe	*V*_60_, cc	0.2±0.6 (38)	0.2±0.6 (38)	0.2±0.5 (38)
Mandible	*V*_55_, %	2.4±4.2 (39)	2.4±4.4 (39)	2.4±4.1 (39)
Left parotid gland	*D*_mean_, Gy	16.0±16.5 (29)	15.9±16.6 (29)	15.8±16.6 (29)
Right parotid gland	*D*_mean_, Gy	16.2±16.5 (27)	16.0±16.6 (27)	15.8±16.5 (27)
Left inner ear	*D*_mean_, Gy	22.7±15.7 (25)	22.5±15.7 (26)	22.5±15.7 (26)
Right inner ear	*D*_mean_, Gy	22.2±14.6 (25)	21.8±14.6 (26)	21.5±14.5 (26)
Oral cavity	*D*_mean_, Gy	32.0±14.0 (40)	31.6±13.8 (40)	31.3±13.6 (40)

The numbers in brackets counted the plans satisfying the clinical requirements

Table 4 summarizes the *D*_mean_ differences and γ passing rates of the air cavities and bony structures in the targets for all forty patients. The *D*_mean_ to the air cavities was underestimated by 1.6% using AAA and by 0.2% using AXB, and the *D*_mean_ to the bony structures was overestimated by 2.3% using AAA and by 0.4% using AXB with respect to the benchmark MC values. The γ passing rates of AXB were higher than those of AAA, indicating that the doses to the air cavities and bony structures in the targets calculated by AXB were more accurate than those calculated by AAA.

**Table 4:** *D*_mean_differences and γ passing rates of the air cavities and bony structures

	V (cc)	Diff (%)			γ (%)		
		AAA	AXB	*p *value	AAA	AXB	*p *value
Air cavities	36.7±36.1	-1.6±0.5	-0.2±0.6	<0.05	86.6±9.4	98.0±1.7	<0.05
Bony structures	41.2±37.9	2.3±0.7	0.4±0.4	<0.05	82.7±13.5	99.0±1.7	<0.05

Diff = (*D*_mean_(TPS) –*D*_mean_(MC))/ *D*_mean_ (MC) ×100%

### Correlation analysis

**Fig. 1:**
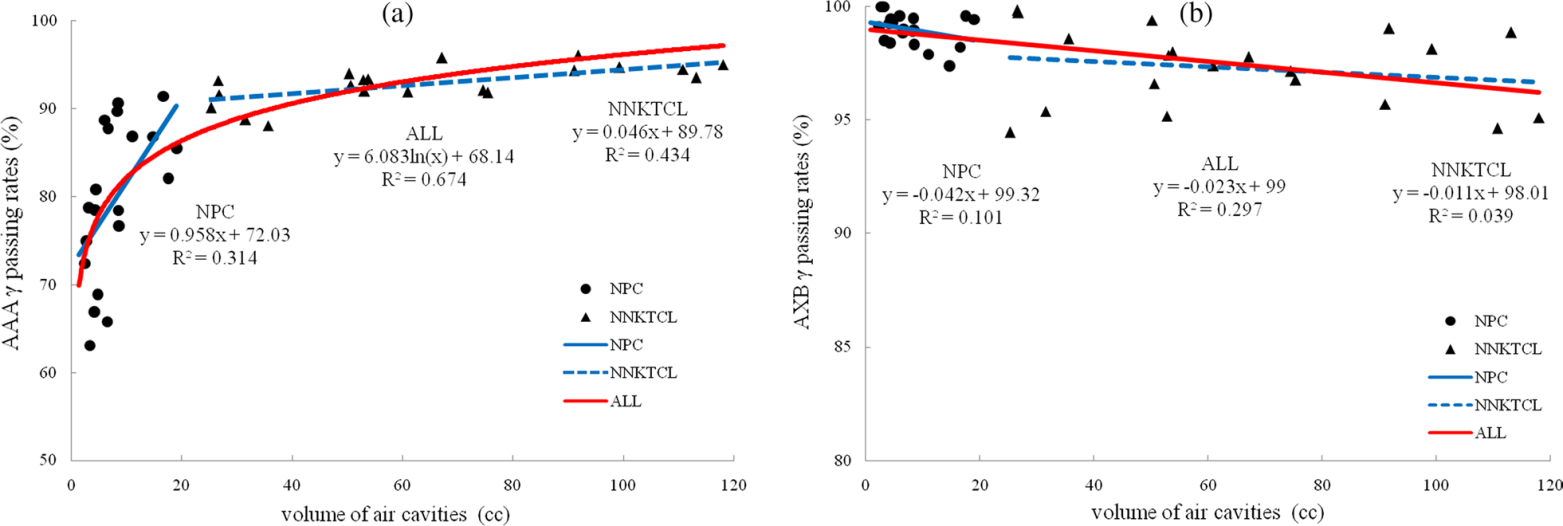
Scatter plots with fitted curves for γ passing rates and *V*_air_using AAA (**a**) and AXB (**b**)

**Fig. 2:**
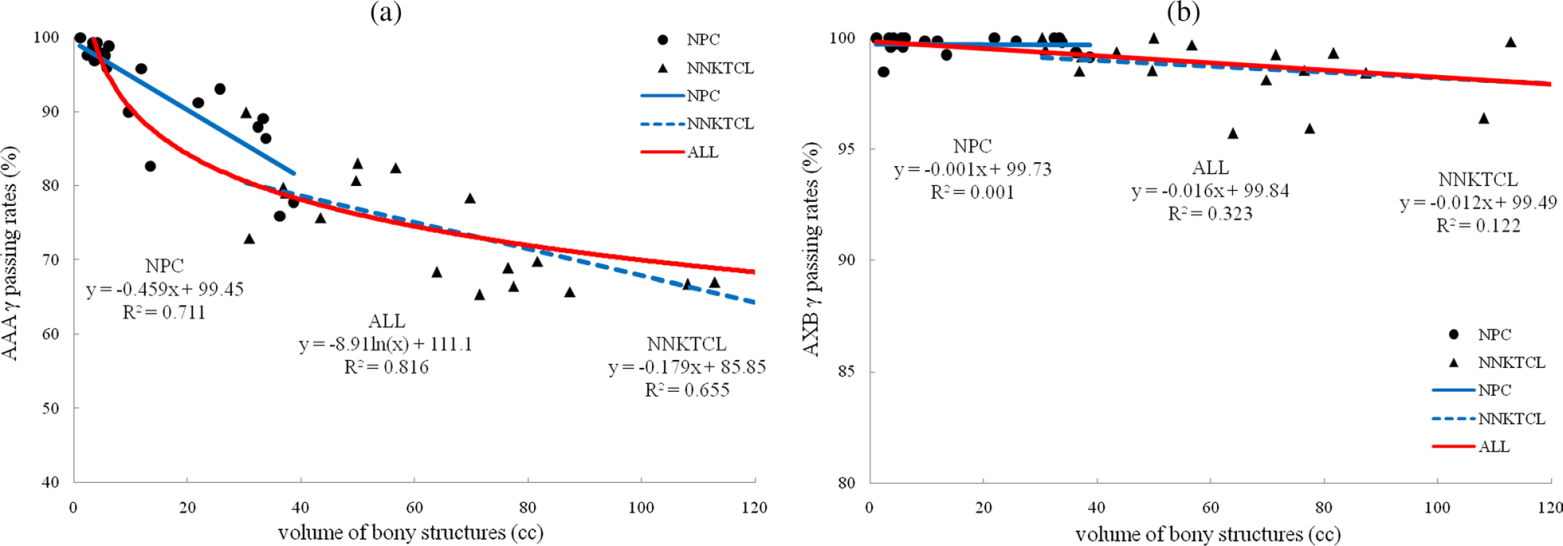
Scatter plots with fitted curves for γ passing rates and *V*_bone_ using AAA (**a**) and AXB (**b**)

Figures 1 and 2 show scatter plots with fitted curves for the γ passing rates using AAA and AXB versus *V*_air_ and *V*_bone_. The *V*_air_ and *V*_bone_ of NPC were smaller than those of NNKTCL. It can be seen from Figs. 1a and 2a that, using AAA, the γ passing rates were proportional to the natural logarithm of *V*_air_ (R^2^ = 0.674) and inversely proportional to the natural logarithm of *V*_bone_ (R^2^ = 0.816). For the 20 NPC patients and 20 NNKTCL patients assessed using AAA separately, the R^2^ values were 0.314 and 0.434 for the air cavities and 0.711 and 0.655 for the bony structures, respectively, which were less than the R^2^ values when the volume were regarded as a whole. The small R^2^ values of AXB are showed in Figs. 1b and 2b, indicating a negligible correlation between the γ passing rates and *V*_air_/*V*_bone_.

## Discussion

Several studies have reported that 5% changes in the dose calculation may result in 20% changes in the local tumour control probability and 30% changes in the normal tissue complication probability [15, 16]. Accurate dose calculations are fundamental for radiotherapy treatment planning and it has been suggested that the error of dose calculation be less than 3% [27]. Thus, it is essential to implement patient-specific dose recalculation QA to ensure acceptable calculation results by the TPS. The MC method was used for our dose recalculation QA and taken as the benchmark to investigate the dose distributions of head and neck IMRT plans produced by AAA and AXB. Finally, the correlation between the γ passing rates and *V*_air_/*V*_bone_ in targets was explored based on our QA results.

Following the reporting and integration of AXB into the Eclipse TPS [28], studied have investigated and compared the calculation results provided by AAA and AXB. For a single field in heterogeneous media, AXB performed better than AAA due to better optimizations for the lateral electronic disequilibrium effect [29–31]. However, the effect was compensated when clinical IMRT plans are created with multiple fields from different directions, so the difference between AAA and AXB may not be obvious. Other experiments demonstrated that both algorithms produced acceptable accuracy with respect to the measured data [32–35]. However, dosimetric measurements introduced several challenges, such as the position of measurement and the particle disequilibrium caused by the inserted material [36].

Some investigations have revealed the dosimetric differences between AAA and AXB in real patients with head and neck cancers [19, 34, 35], but these differences need to be benchmarked against the gold standard, the MC method. AXB algorithm and Monte Carlo method can both report the absorbed dose in two modes: dose to medium and dose to water. Han et al. [33] reported verification results for AXB using the Radiological Physics Center head and neck phantom. The dose distributions predicted by AXB with both dose to medium and dose to water modes were compared to the doses measured using thermo luminescent dosimeters and films. The authors observed that the dose to medium mode produced slightly better agreement with the measurement results than the dose to water mode. Ma et al. [37] suggested that to achieve consistency with previous radiation therapy experiences, MC photon algorithms should report using dose to medium mode for treatment plan evaluation and treatment outcome analysis. Therefore, AXB and MC algorithms were configured to report in the dose to medium mode in our research.

Our patient-specific dose recalculation QA results showed that the target coverage produced by AXB had better agreement with MC than AAA. However, the prescribed dose coverage of PGTVnx and PTV produced by AXB were 1.2% and 2.5% lower, respectively, than that produced by AAA, which was expected according to the results of previous studies [19, 34, 35]. The γ passing rates of AAA and AXB for PGTVnx and PTV were statistically significant because these regions presented with many air cavities and bony structures, affecting accurate dose calculations. PTV1 contained PGTVnx, so the γ evaluation of AAA and AXB for PTV1 also showed statistical significance.

It should be noted that in this study, the *D*_max_ values of the targets and serial OARs (brain stem, spinal cord, optic chiasm, optic nerves, pituitary and lens) predicted by AXB were all greater than those predicted by AAA, which was not consistent with previous literature [19, 34, 35]. A *D*_max_ of the target exceeding 110% of the prescribed dose would be moderately acceptable; however, the calculation of different *D*_max_ values for serial OARs by different algorithms requires dose recalculation QA checks to ensure patient safety. It is more acceptable and reasonable to assess clinical plans with *V*_110%_ for high dose regions of targets and *D*_1%_ for serial OARs. In contrast, the *D*_mean_ of parallel OARs (eyes, parotid glands, inner ears and oral cavity) predicted by AXB was smaller than that predicted by AAA, and the *V*_60_ of the temporal lobes and *V*_55_ of the mandible predicted by AXB were equal to those predicted by AAA. Using AAA and AXB, the γ values of the mandible and oral cavity were all > 95%, which attracting less attention than the targets and priority 1 OARs [38]. However, the statistical significance of the γ values for the mandible and oral cavity calculated by both AAA and AXB also reflected the different performances of AAA and AXB in the air cavities and bony structures.

Previous investigations have observed better agreement between AXB and MC within extremely low or high density materials [29–31]. Our study demonstrated that the air cavities and bony structures had an impact on the accurate dose calculation by AAA for both the targets and OARs in clinical IMRT plans. Although the dosimetric parameters produced by AAA tended to satisfy clinical requirements, those produced by AXB and MC were more consistent.

Compared with the MC algorithm, AAA and AXB underestimated the *D*_mean_ inside the air cavities by 1.6% and 0.2% and overestimated the *D*_mean_ inside the bony structures by 2.3% and 0.4%, respectively. Figures 1 and 2 show the relationship between the γ passing rates and *V*_air_/*V*_bone_ more clearly. From the scatter plots for AAA, whether separately or jointly for NPC and NNKTCL, the larger *V*_air_ was or the smaller *V*_bone_ was, the higher the γ passing rate was. However, a negligible correlation was found between the γ values predicted by AXB and *V*_air_/*V*_bone_, indicating that the air cavities and bony structures had little impact on the accurate dose calculation of AXB. The γ passing rates from AAA were proportional to the natural logarithm of *V*_air_ and inversely proportional to the natural logarithm of *V*_bone_. When *V*_air_ in the targets was smaller than approximately 80 cc or *V*_bone_ was larger than approximately 6 cc, the γ values from AAA were below 95%.

The *V*_air_ and *V*_bone_ of NPC were generally smaller than those of NNKTCL because of differences in the target location. Therefore, the curves of the γ values versus *V*_air_/*V*_bone_ for NPC and NNKTCL were fitted separately. NPC and NNKTCL are both head and neck cancers, however, and when they were considered as a whole, higher R^2^ values were obtained for the fitted curves. This suggested that the relationship between the γ values and *V*_air_/*V*_bone_ discovered in this study may be present in other head and neck cancers, but this remains to be further explored.

In addition, we compared 0.5% uncertainty with 2% uncertainty of the MC method to clarify the impact of statistical uncertainty on the relationship between the γ values and *V*_air_ or *V*_bone_. Using 2% statistical uncertainty, the γ values from AAA in the air cavities and bony structures were decreased by 5.7 ± 4.3% and 5.3 ± 4.1%, respectively, and the corresponding γ values from AXB were decreased by 4.5 ± 3.1% and 3.7 ± 2.8%. The dose discrepancies caused by statistical uncertainty were obvious; therefore it is necessary to set the statistical uncertainty of the MC method as small as possible. The accuracy of dose calculation should be traded off for time. However, using 2% statistical uncertainty, the γ values from AAA were still proportional to the natural logarithm of *V*_air_ and inversely proportional to the natural logarithm of *V*_bone_ but with slightly lower R^2^ values, and there remained a negligible correlation between the γ values from AXB and *V*_air_/*V*_bone_.

## Conclusion

To ensure that the deviation between the actual dose given to the patient and the dose distribution calculated by the TPS is within reasonable limits, patient-specific dose recalculation QA must be implemented. The dose discrepancies caused by the air cavities and bony structures need to be considered when using different dose algorithms. In clinical QA practice, the effect of *V*_air_ and *V*_bone_ in the targets on γ passing rates should be considered.

## Data Availability

The datasets used during this study are available from the corresponding author on reasonable request.
